# New sources of soybean seed meal and oil composition traits identified through TILLING

**DOI:** 10.1186/1471-2229-9-89

**Published:** 2009-07-14

**Authors:** Emily C Dierking, Kristin D Bilyeu

**Affiliations:** 1 University of Missouri-Columbia, Division of Plant Sciences, 110 Waters Hall, Columbia, MO 65211, USA; 2 USDA-ARS, Plant Genetics Research Unit, 110 Waters Hall, Columbia, MO 65211, USA

## Abstract

**Background:**

Several techniques are available to study gene function, but many are less than ideal for soybean. Reverse genetics, a relatively new approach, can be utilized to identify novel mutations in candidate genes; this technique has not produced an allelic variant with a confirmed phenotype in soybean. Soybean raffinose synthase genes and microsomal omega-6 fatty acid desaturase genes were screened for novel alleles in mutagenized soybean populations.

**Results:**

Four mutations in independent lines were identified in the raffinose synthase gene *RS2*; two mutations resulted in amino acid mutations and one resulted in an altered seed oligosaccharide phenotype. The resulting phenotype was an increase in seed sucrose levels as well as a decrease in both raffinose and stachyose seed oligosaccharide levels. Three mutations in independent lines were identified in the omega-6 fatty acid desaturase gene *FAD2-1A*; all three mutations resulted in missense amino acid mutations and one resulted in an altered seed fatty acid profile that led to an increase in oleic acid and a decrease in linoleic acid in the seed oil.

**Conclusion:**

The oligosaccharide phenotype controlled by the novel *RS2 *allele is similar to previously observed seed oligosaccharide phenotypes in *RS2 *mutant (PI 200508) allele-containing lines. Due to the anti-nutritional characteristics of raffinose and stachyose, this represents a positive change in seed composition. The fatty acid phenotype controlled by the novel *FAD2-1A *allele controls an increase in oleic acid in the seed oil, a phenotype also observed in a line previously characterized to have a null allele of the *FAD2-1A *gene. Molecular marker assays were developed to reliably detect the inheritance of the mutant alleles and can be used in efficient breeding for these desired seed phenotypes. Our results serve as the first demonstration of the identification of soybean mutants controlling seed phenotypes discovered through the reverse genetics technique TILLING.

## Background

Reverse genetics is potentially a powerful strategy used to identify novel, induced mutations in candidate genes. Utilizing reverse genetics allows us to take advantage of genes characterized in other plant genomes and use this knowledge to create a pool of candidate genes in soybean which can then be screened for genotypic variants. The function of the gene is further characterized by identifying predicted mutant phenotypes. Alternatively this technique can be applied to genes with known function to create an allelic series to avoid possible lethality issues in genes essential for plant growth or development. The recent release of the soybean genome sequence is an especially valuable asset for soybean reverse genetics.

Targeting Induced Local Lesions IN Genomes (TILLING) is a reverse genetics technique that serves as a high throughput method to identify unique, chemically induced mutations within target genes which have potential to change gene expression and/or function [1-3]. We developed two soybean TILLING populations utilizing the chemical mutagen ethyl-methanesulfonate (EMS) as part of an effort to provide a resource to identify novel alleles of genes with a role in soybean seed composition traits [4]. Chemical mutagenesis, either EMS or NMU (N-nitroso-N-methylurea) typically induces single nucleotide polymorphisms; these point mutations are extremely useful both for studying gene function as well as for their potential use in crop improvement [4]. The mutants are generally characterized by knocked-down or altered gene function rather than a knock-out; for the populations used in this study, the previously characterized mutation distributions were 45 or 33% missense, 51 or 58% silent, and 4 or 8% truncation [4].

Previously, a number of techniques which were first proven in other crops have been applied to soybean functional genomics studies, but each strategy has obstacles in terms of efficiency, directness, timing, and regulatory issues. Transformation of soybean with either *Agrobacterium tumefaciens *or *A. rhizogenes *has been effective in studying gene function, although it is not very efficient [5] and can be limited by genotype specificity [6]. Directed co-suppression, overexpression of genes, and RNAi have all been utilized to gain insights into gene function and produce desired phenotypes in transgenic soybeans [7-10]. While the use of transgenic technology is suitable for traits that can be utilized broadly for commodity soybeans, the current regulatory environment is prohibitive to extensive use of transgenic technology for many individual soybean traits.

The more traditional forward genetics approach relies on the identification of a mutant phenotype followed by the investigation of the causative gene. Forward genetics screening has historically been a very valuable strategy to identify different sources of soybean traits. However, screening divergent germplasm or induced mutant populations may fail to deliver the desired phenotype when multiple genes are involved. In soybean, the genome functions as diploid, but it has undergone rounds of genome duplication that often results in functional genetic redundancy [11-15].

The objective of this work was to take advantage of the availability of soybean candidate genes that were known to control seed composition traits as targets for the TILLING reverse genetics strategy; the goal was to identify novel mutant alleles that could be characterized for desirable seed phenotypes. Our targets were a raffinose synthase (EC 2.4.1.82) and an omega-6 fatty acid desaturase (EC 1.3.1.35) controlling seed oligosaccharide levels and oleic acid levels in the seed oil, respectively.

Raffinose synthase catalyzes the biochemical reaction to produce raffinose from sucrose and galactinol. Stachyose is formed in a stepwise reaction utilizing raffinose and galactinol as substrates. Both raffinose and stachyose are indigestible by monogastric animals and are therefore considered anti-nutritional components of soybean meal. Previously, the PI 200508 allele of *RS2 *was associated with the increased sucrose and low raffinose and stachyose seed phenotype [16].

In addition to the targeted raffinose synthase candidate gene, we screened for novel mutant alleles of the soybean seed-expressed omega-6 fatty acid desaturase gene *FAD2-1A*. This fatty acid desaturase catalyzes the conversion of oleic acid precursors into linoleic acid precursors that accumulate in the seed oil [17,18]. Mutations in *FAD2-1A *have been recovered previously using a forward genetics strategy, and result in an increase in oleic acid levels and a decrease in linoleic acid levels, a phenotype desirable for cooking and industrial oils. The elevated oleic acid soybean line M23 was induced by X-ray mutagenesis and has been shown to have a genomic deletion that includes the *FAD2-1A *gene; a second elevated oleic acid soybean line contained a single base deletion in the *FAD2-1A *gene [18,19]. Cooking oil is in demand that contains elevated oleic acid and decreased linolenic acid. Although the genetic combining ability for mutant alleles of the *FAD2-1A *gene and microsomal omega-3 fatty acid desaturase (*FAD3*) genes has been reported, the exact relationship between increased oleic acid levels and decreased linolenic acid levels has not been clearly defined [[20,21] K. Bilyeu, unpublished]. The oleic acid level in soybean seed oil has been demonstrated to be very sensitive to the environment [22], which has complicated the identification and analysis of elevated oleic acid soybean lines.

## Results

### Identification of soybean raffinose synthase (RS2) mutant alleles

**Figure 1:**
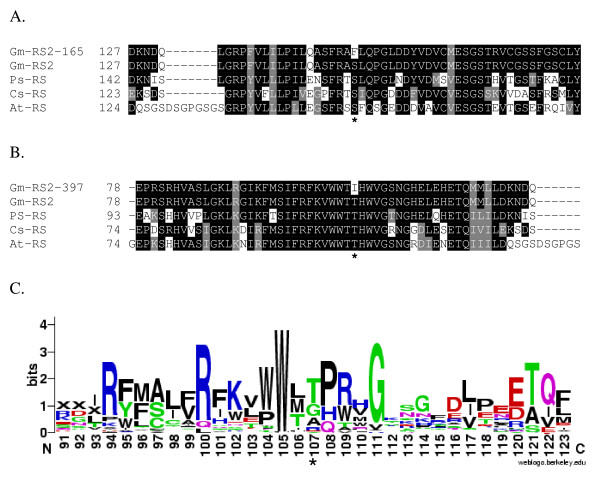
**Raffinose synthase amino acid sequence alignments in the regions surrounding the induced mutations in the *RS2 *gene**. Amino acid positions are indicated at the beginning of each alignment. The position of the polymorphic amino acid is indicated by an asterisk. Identical amino acid residues are highlighted in black while similar amino acid residues are highlighted in gray. A. The exon one region containing the induced mutation in line 165 which resulted in S150F. B. The exon one region containing the induced mutation in line 397 which resulted in T107I. C. Weblogo output of the amino acid conservation of raffinose synthase enzymes aligned as part of the BLINK feature at NCBI  using GI number 187610414. Amino acid positions within the protein are listed on the X axis. The overall height for each amino acid column stack indicates the sequence conservation at that position while the height of one-letter amino acid symbols within the column stack indicates the relative frequency of each amino acid in that position [23].

Previously, mutations in the soybean raffinose synthase gene, *RS2*, have been shown to result in an increase in seed sucrose and a decrease in raffinose and stachyose [16]. Reverse genetics screening of the EMS mutagenized populations created the potential to find additional mutations in *RS2 *and confirm the contribution of this gene to the seed oligosaccharide phenotype in soybean. A portion of the *RS2 *gene [GenBank: EU651888] was screened for mutations utilizing the TILLING strategy [4]; four lines were identified which contained single nucleotide polymorphisms (SNPs). These lines were subsequently confirmed by sequence analysis to contain independent *RS2 *mutations. The four identified lines all contained a SNP typical of EMS mutagenesis, G/C to A/T transitions. Two of the mutations did not result in amino acid changes and therefore were not considered candidates for phenotypic characterization. The other two lines, designated 165 and 397, contained mutations which resulted in missense amino acid changes (Figure 1)[23].

DNA from M_2 _tissue of line 165 contained a homozygous SNP (c448t in the coding sequence) resulting in S150F amino acid change. DNA from M_2 _tissue of line 397 contained a heterozygous SNP (c319t in the coding sequence) resulting in a T107I amino acid change. The induced mutations in both line 165 and 397 lie in semi-conserved regions of plant raffinose synthase gene sequences (Figure 1). M_3 _seedlings from lines 165 and 397 were characterized for the *RS2 *alleles, and the homozygous and segregating nature of the identified mutant alleles was confirmed, respectively.

### Identification of omega-6 fatty acid desaturase FAD2-1A mutant alleles

Mutations in the soybean omega-6 fatty acid desaturase gene *FAD2-1A *have been shown to elevate oleic acid content of the seed oil [18,19]. Therefore, one of our main targets was to identify variant alleles of the *FAD2-1A *gene present in the TILLING populations. Primers were designed to specifically amplify and interrogate the *FAD2-1A *sequences present in the mutant populations, and three individual lines were identified; these lines were subsequently confirmed by sequence analysis to contain independent *FAD2-1A *mutations.

**Figure 2:**
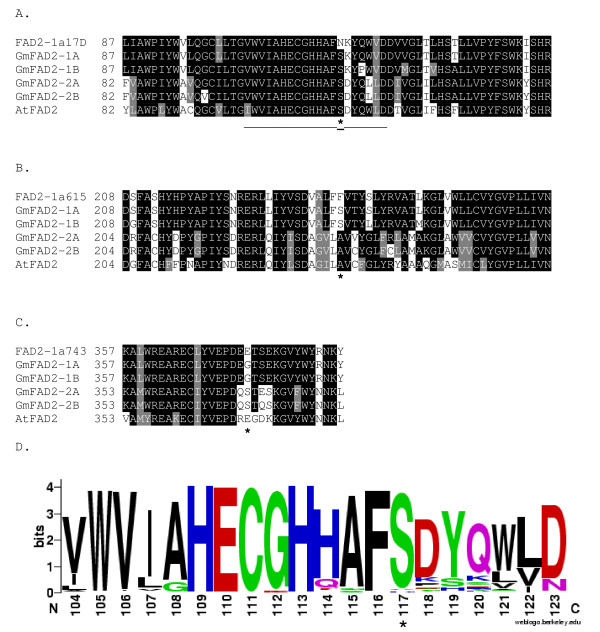
**Omega-6 fatty acid desaturase (FAD2) amino acid sequence alignments in the regions surrounding the induced mutations in the soybean *FAD2-1A *gene**. Amino acid positions are indicated at the beginning of each alignment. The position of the polymorphic amino acid is indicated by an asterisk. Identical amino acid residues are highlighted in black while similar amino acid residues are highlighted in gray. Underlined amino acids represent the histidine-rich region Ia, a critical region for fatty acid desaturase enzyme function [24]. A. The region containing the induced mutation in line 17D which resulted in S117N. B. The region containing the induced mutation in line 615 which resulted in S238F. C. The region containing the induced mutation in line 743 which resulted in G374E. D. Weblogo output of the amino acid conservation in region Ia of omega-6 fatty acid desaturase enzymes [24] aligned as part of the BLINK feature at NCBI using GI number 197111724.

The *FAD2-1A *mutations identified in DNA from M_2 _tissue were homozygous in all three cases, and the mutations were confirmed in the M_3 _seedlings corresponding to the original M_2 _plants. Line 17D contained a SNP (g350a in the coding sequence) resulting in the amino acid change S117N. Line 615 contained a SNP (c713t in the coding sequence) resulting in the amino acid change S238F. Line 743 contained a SNP (g1121a in the coding sequence) resulting in the amino acid change G374E. The missense mutation for line 17D was in a highly conserved region of the protein sequence, while the missense mutations in lines 615 and 743 were in less conserved regions (Figure 2)[24].

### Oligosaccharide content phenotype of RS2 induced mutants

Seeds from the homozygous S150F line 165 did not have an obvious oligosaccharide phenotype as determined by quantitatively measuring sucrose, raffinose and stachyose of M_3 _seeds and comparing them to wild-type seeds (data not shown). However, the line 397 harboring the T107I *RS2 *allele displayed a phenotype predicted for mutations in the soybean raffinose synthase gene *RS2*.

**Figure 3:**
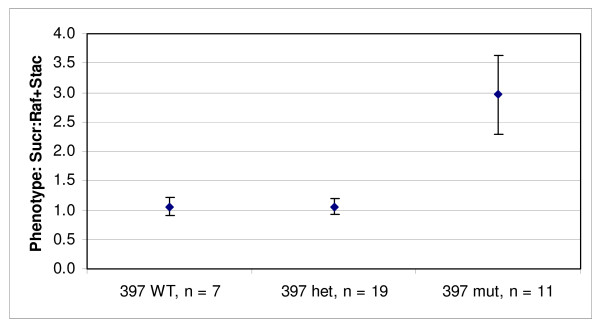
**Phenotype to genotype association of segregating M_3 _seeds from line 397**. The x-axis is represented by the three distinct *RS2 *genotypes: WT represents wild-type *RS2 *alleles, mut represents mutant, and het represents heterozygous *RS2 *alleles from the line 397; n is the number of individual seeds for each class. The oligosaccharide phenotype of 37 individual M_3 _seeds was determined. The data represents the mean of the ratio of extractable seed sucrose to the sum of raffinose and stachyose. Error bars represent plus and minus one standard deviation from the mean.

Taking advantage of the heterozygous state of the induced mutation in line 397, we investigated the inheritance of this novel allele and its subsequent effect on seed oligosaccharide content by screening thirty-seven individual M_3 _seeds for both oligosaccharide phenotype and *RS2 *genotype (Figure 3). Seeds were chipped into two approximately equal pieces, one was used for single seed oligosaccharide phenotype analysis and the remaining portion containing the embryo was germinated and genotyped by the developed allele specific molecular marker assay. The genotype/phenotype association results on M_3 _seeds reveal an increase in sucrose along with decreases in raffinose and stachyose content when seeds were homozygous for the mutant *RS2 *allele (Figure 3). Furthermore, one wild-type allele of *RS2 *was sufficient to produce the wild-type oligosaccharide seed phenotype, which is consistent with previous results (Figure 3) [16].

**Figure 4:**
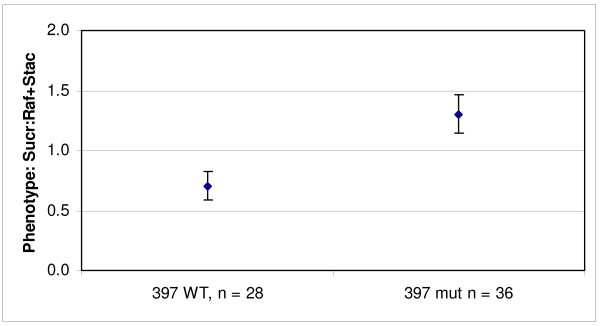
**Phenotype to genotype association of a homozygous soybean population derived from the mutagenized soybean line 397**. The x-axis is represented by two distinct *RS2 *genotypes: WT represents wild-type *RS2 *alleles and mut represents mutant *RS2 *alleles from the line 397; n represents the number of individual seeds from each genotypic class. The oligosaccharide phenotype of four individual M_4 _seeds from each plant was determined. The data represents the mean of the ratio of extractable seed sucrose to the sum of raffinose and stachyose. Error bars represent plus and minus one standard deviation from the mean.

A population consisting of plants with contrasting *RS2 *genotypes was then developed from line 397-derived plants that contained either homozygous wild-type (Williams 82) or homozygous mutant alleles at the *RS2 *locus in order to further characterize the phenotype resulting from the novel allele. Seven independent wild-type *RS2 *and nine independent mutant *RS2 *plants were selected to negate the action of unidentified genes that may contribute to the oligosaccharide content; the mutation density was previously determined to average 1/550 kilobases [4]. Four seeds from each of the plants of the homozygous population were analyzed for oligosaccharide content. For the plants that contained the T107I *RS2 *mutation, the average seed sucrose was increased by 28%, raffinose was reduced to 37% and stachyose was reduced to approximately 24% of 397-derived seeds which carried the wild-type allele of *RS2 *(Figure 4). This oligosaccharide phenotype is similar to the phenotype controlled by the previously described *RS2 *mutant alleles in PI 200508 which also resulted in a decrease in seed raffinose and stachyose content along with an increase in seed sucrose levels [16].

In all cases where *RS2 *was homozygous mutant, a statistically significant difference in the ratio of sucrose to the sum of raffinose and stachyose was observed when compared to seeds with either *RS2 *homozygous wild-type or heterozygous alleles. The absolute ratios observed in the homozygous classes were significantly different between the segregating seed samples (Figure 3) and the homozygous plants analyzed (Figure 4); a higher mean ratio was observed for the field grown seeds compared to the seeds produced in the growth chamber.

### Fatty acid phenotype of FAD2-1A mutants

**Figure 5:**
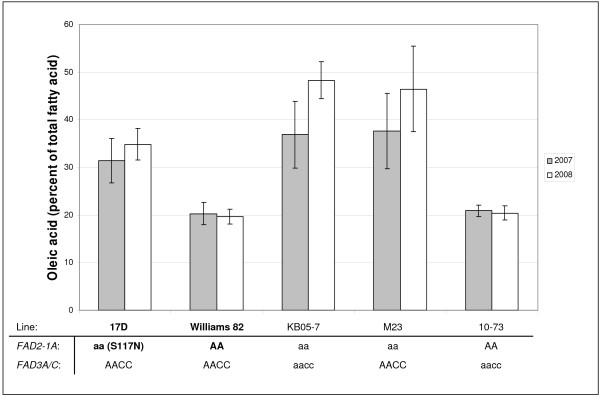
**Increased oleic acid content in soybean mutant *FAD2-1A *mutant lines compared to *FAD2-1A *wild-type lines from field produced seeds in two years**. Histograms represent the mean oleic acid content as a percentage of the seed oil for 25 individual seed samples representing five plants from each line listed on the x-axis. Error bars represent plus and minus one standard deviation of the mean. For each line, the *FAD2-1A, FAD3A*, and *FAD3C *genotypes are listed below the line name; lowercase letters represent the mutant case, and uppercase letters represent the wild-type case. Line 17D is the result of mutagenesis of line Williams 82, and contains an S117N missense mutation of *FAD2-1A *(bolded for comparison); M23 contains a genomic deletion of *FAD2-1A *[18]; 10–73 contains mutant alleles of *FAD3A *and *FAD3C*, which reduce linolenic acid levels but do not affect oleic acid levels [33]; KB05-7 is a derivative of a cross between 10–73 and M23 which combines mutant alleles of *FAD2-1A*, *FAD3A*, and *FAD3C*.

The expected phenotype for soybeans containing mutations in *FAD2-1A *is an increase in the oleic acid content of the seed oil with a concomitant decrease in linoleic acid. Since the mutant lines were homozygous for the mutations, all three lines were grown two years in a field environment along with lines that contained wild-type or mutant alleles of *FAD2-1A*, *FAD3A *and *FAD3C *to produce seeds for fatty acid analysis. Lines 615 and 743 did not produce significantly different oleic acid levels from the progenitor line 'Williams 82' [25] and were only grown in 2007 (data not shown). Oleic acid levels for line 17D containing homozygous *FAD2-1A *S117N mutant alleles were significantly higher than those for Williams 82 (Figure 5). The mean oleic acid level for the seeds containing homozygous *FAD2-1A *17D mutant alleles was lower than the mean oleic acid level for two independent lines which possess a null *FAD2-1A *allele (KB05-7 and M23, Figure 5). The results were similar for the experiments performed in 2007 and 2008, although there was a trend for higher oleic acid levels in 2008 for the lines containing *FAD2-1A *mutations.

**Figure 6:**
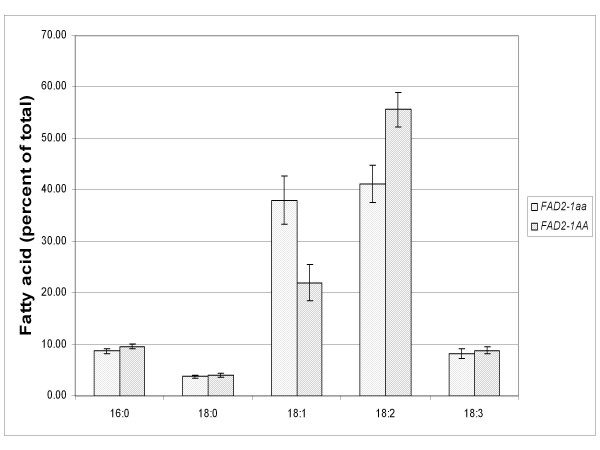
**Inheritance of S117N alleles of *FAD2-1A *results in increased mean oleic acid and decreased linoleic acid soybean seed oil**. Soybean lines with contrasting homozygous mutant or wild-type *FAD2-1A *alleles were developed from a cross of line 17D with Williams 82. Homozygous mutant S117N *FAD2-1A *lines (*FAD2-1aa*) and homozygous wild-type (*FAD2-1AA*) lines were grown in the field in 2008. Fatty acid profiles were determined for individual seeds and histograms of mutant (spotted) or wild-type (diagonal lines) represent the mean relative palmitic acid (16:0), stearic acid (18:0), oleic acid (18:1), linoleic acid (18:2), and linolenic acid (18:3) content of the oil. Error bars represent plus and minus one standard deviation of the mean.

The inheritance of the S117N mutant *FAD2-1A *allele from line 17D and the fatty acid phenotype was evaluated in selected homozygous progeny derived from a cross of Williams 82 with line 17D. Molecular marker assays specific for the S117N mutant *FAD2-1A *allele from 17D were designed and validated. Six independent F_3 _plants homozygous for the 17D mutant alleles and five independent homozygous wild-type plants were grown to produce seed in a field environment. Fatty acid analysis on the resulting F_4 _seeds demonstrated significantly higher mean oleic acid levels for those seeds which were homozygous for the mutant S117N allele of *FAD2-1A *(Figure 6). The mean linoleic acid level for the *FAD2-1A *mutant seeds was significantly lower than the wild-type *FAD2-1A *seeds. Overall, the significant change in oleic acid content and the concomitant decrease in linoleic acid content is consistent with the S117N alleles of *FAD2-1A *responsible for disrupting at least part of the seed expressed omega-6 fatty acid desaturase enzymatic capacity.

## Discussion

Here we demonstrate the utility of the TILLING reverse genetics strategy in identifying novel soybean lines containing desirable seed traits. The targeted raffinose synthase and omega-6 fatty acid desaturase genes were chosen based on previous research revealing their involvement in seed oligosaccharide content and oleic acid levels, respectively. For both genes, multiple variant alleles were identified, and one line containing mutant alleles of each of the genes produced the predicted phenotype. The phenotypes were confirmed to be dependent on the inheritance of the mutant alleles. Because the roles of the candidate genes were confirmed, the developed molecular marker assays enable direct selection for the mutant allele in the heterozygous state, when the phenotype would not otherwise be apparent. Seed mutagenesis and reverse genetics are not transgenic technologies, so soybean varieties that incorporate mutant alleles identified by TILLING are conventional lines which are not subject to any regulatory restrictions. Soybean traits identified by TILLING can be rapidly incorporated into elite soybean cultivars and released to producers.

The identification of an induced mutation in *RS2 *serves as an additional confirmation of the contribution of this gene to the seed raffinose and stachyose content [16]. Similar to the PI 200508 mutant allele of *RS2*, the mutagenized line 397 has reduced raffinose and stachyose as well as an increase in seed sucrose content. Since similar phenotypes resulted from two independent mutations in *RS2*, it may indicate that the mutations in the gene are deleterious to enzyme function and the detected raffinose in these lines is the result of one or more additional soybean raffinose synthase genes. It appears that inheritance of a single wild-type allele of *RS2 *is sufficient for a wild-type oligosaccharide seed phenotype, which is consistent with previously characterized *RS2 *alleles [16].

Soybean seed oligosaccharide content appears to be controlled mainly by the *RS2 *gene. However, both raffinose and stachyose are still present in lines containing *RS2 *mutants, suggesting that additional raffinose synthase enzyme activity remains during seed development. Other candidate raffinose synthase genes have been identified, but variant alleles have not been confirmed to be associated with altered oligosaccharide content. It is possible that combining an *RS2 *mutation with variant alleles of other raffinose synthases may reveal epistatic interactions that would otherwise have been masked by a wild-type version of *RS2*.

The novel S117N allele of *FAD2-1A *appears to be deleterious to enzyme function since oleic acid accumulates to significantly higher levels in the seed oil in lines homozygous for the mutation when compared to related lines containing wild-type *FAD2-1A*. Since the environment has been shown to have an effect on oleic acid content, it was not surprising that the standard deviations were high for oleic acid content for the investigated *FAD2-1A *mutant TILLING line 17D and the *FAD2-1A *deletion lines M23 and KB05-7 [22]. The observed differences in means with overlapping standard deviations for oleic acid phenotypes and the differences in relative maturity among the TILLING mutant line with the novel *FAD2-1A *alleles and the *FAD2-1A *deletion lines M23 and KB05-7 indicates more research will be necessary to clarify the extent of the phenotypic consequences of the *FAD2-1A *S117N substitution. Nevertheless, this novel *FAD2-1A *mutant allele provides an additional resource to investigate the agronomic impact of the elevated oleic acid trait in soybeans and an independent confirmation of the contribution of the *FAD2-1A *gene to oleic acid accumulation in soybean seeds.

Convincing evidence exists that points to genetic redundancy playing a role in oleic acid accumulation. Although the identification of independent *FAD2-1A *alleles has demonstrated a role for the gene in oleic acid accumulation, the residual omega-6 fatty acid desaturase activity that allows the production of linoleic and linolenic acids may be encoded by one other closely related gene (*FAD2-1B*) which has been shown to be expressed in soybean seeds or the *FAD2-2 *gene family members members [11,19,26-28]. A reverse genetics strategy is particularly amenable to dissection of desirable phenotypes where genetic redundancy may complicate a forward genetics strategy.

## Conclusion

The use of TILLING to identify novel sources of important soybean seed composition traits confirms the utility of this reverse genetics technology as a route for both gene function analysis and direct applicability for soybean improvement. Soybean now joins wheat, sorghum, pea, and rapeseed as crops that have demonstrated success in identifying traits using TILLING for reverse genetics [29-32].

## Methods

### Population Development

The 'Williams 82' [25] EMS mutagenized populations screened in this study were previously described [4]. The populations screened were exposed to 40 or 50 mM EMS. M_1 _plants were advanced to M_2 _families, leaf tissue was collected and DNA prepared from a single M_2 _plant from each family. M_3 _seeds from each M_2 _plant were catalogued for storage.

### Development of *RS2 *contrasting lines

Thirty-nine seeds were planted in packets (CYG, Mega International, St. Paul, MN), allowed to germinate, and transferred to soil in flats. Plants were sampled for genotypic determination by allele-specific molecular marker assay described below. A population of only the plants homozygous for either the wild-type or mutant allele of *RS2 *were transplanted to 3-gallon pots; 1–3 plants per pot. Seven homozygous wild-type and nine homozygous mutant *RS2 *M_2:3 _plants from the mutagenized line 397 were grown to maturity in a growth chamber with 13 hour day length. The dark temperature was 22°C and the light temperature was 28°C. Plants were grown, three per 3-gallon pot, in PRO-MIX (Premier Horticulture) medium and fertilized with Osmocote Plus (Scotts) per manufacturer's instructions.

### Field plant growth

Plants for seed fatty acid phenotype determination were grown at the Bradford Research and Extension Center (BREC) located near Columbia, MO in the summer of 2007 and 2008 with irrigation as needed. Williams 82 was the control line since it was the progenitor of line 17D. Several other lines were grown for comparison because of their known mutant alleles in the *FAD2-1A, FAD3A*, and *FAD3C *genes: M23 contains a deletion of *FAD2-1A *[18]; 10–73 contains mutant alleles of *FAD3A *and *FAD3C *[33]; KB05-7 is a combination of mutant *FAD2-1A, FAD3A*, and *FAD3C *alleles from M23 and 10–73 (K. Bilyeu and J. Shannon, unpublished).

### Development of *FAD2-1A *contrasting lines

Seeds of a Williams 82 × 17D cross were produced at BREC in 2007. The F_1 _seeds were advanced to F_2_:_3 _lines in a Costa Rica winter nursery. Two F_3 _seeds from each line were germinated and evaluated for their *FAD2-1A *genotype using the molecular marker assay. Homozygous wild-type or mutant S117N *FAD2-1A *lines were confirmed by analyzing three additional seedling genotypes from each line. Six individual homozygous mutant *FAD2-1A *F_3 _plants representing two independent F_2 _individuals and five individual homozygous wild-type *FAD2-1A *F_3 _plants representing two independent F_2 _individuals were germinated in germination packets and transferred to the field at BREC in 2008 for production of F_4 _seeds. Five seeds from each plant were sampled for individual fatty acid analysis.

### Population Screening

#### Reverse genetics gene screening

A portion of *RS2 *was screened for EMS induced mutations. Exon 1 of *RS2 *was screened using IR 700 and IR 800 labeled primers: 5'-GAGTCTCATATTGTACATGGTAG-3' and 5'-GCAATTCGATGCTTCTTATGAG-3'. A portion of *FAD2-1A *was first amplified with unlabeled primers (to overcome poor amplification of DNA directly with the labeled primers) followed by amplification with IR 700 and IR 800 labeled primers: 5'GTAGAGGTCGTGTGGCCAAAGTGGAAG-3' and 5'AACCATGATCGCAACAAGCTGTTTCAC-3'. Standard TILLING PCR parameters were as follows: One cycle of 95°C for 2 minutes and 94°C for 20 seconds followed by 56 cycles of 94°C for 20 seconds, 56°C for 30 seconds, and 72°C for 1 minute. The next step in the PCR was 72°C for 5 minutes, then a 99°C step for 10 minutes followed by a 70°C to 0°C melt. The reactions were then held at 10°C. Cel I based cleavage of PCR products and detection with polyacrylamide gels was essentially as described [2].

Pools containing cleaved products indicating an induced mutation or heteroduplex mismatch were deconvoluted by separating the pools into individual plant DNA samples for sequencing in order to identify the line containing the mutation [1-3]. The location of the mutation as well as the zygosity could then be verified. The *RS2 *mutations were confirmed by PCR amplification of a portion of the gene followed by sequence analysis. Primers used were 5' CCCACCATGTCACCACACC-3' and 5'-GGTGATGAATTTTTAGCGGCG-3'. PCR parameters were 95°C for 10 minutes, followed by 35 cycles of 95°C for 30 seconds, 60°C for 30 seconds, and 72°C for 30 seconds, and then 5 minutes at 72°C; the reaction was held at 4°C. Screening for the *RS2 *candidate gene was carried out at Purdue University in West Lafayette, IN and screening for the *FAD2-1A *candidate gene was carried out at the Fred Hutchinson Cancer Research Center.

### Allele-Specific Molecular Marker Assay Development

#### RS2 allele-specific molecular marker assay

An allele specific molecular marker assay was developed for the mutation identified in line 397 to discriminate between wild-type Williams 82 or mutant alleles of the *RS2 *gene. The assay was designed as described [34]. In order to achieve allele specificity, single base pair mismatches were introduced into the primer sequence to increase the discriminatory power of the allele-specific primer. These bases and the tails are indicated in lowercase in the primer sequences. Primer sequences were: 5'-gcgggcGTTGCTACCGACCCAGtGAA-3', 5'-gcgggcagggcggcGTTGCTACCGAC CCAGcGAG-3', and a common forward primer 5'-CAGAGGAATAAAATTCATGAGCATA-3'.

Reactions were carried out in 20 μl; each primer was at 0.5 μM final concentration in reactions containing template, buffer (40 mM Tricine-KOH (pH 8.0), 16 mM KCl, 3.5 mM MgCl_2_, 3.75 μg ml^-1 ^BSA, 200 μM dNTPs), 5% DMSO, 0.25× SYBR Green I, and 0.2× Titanium *Taq *polymerase (BD Biosciences, Palo Alto, CA).

PCR parameters on a DNA Engine Opticon 2 (MJ Research/Bio-Rad, Hercules, California) for the *RS2 *assay were as follows: 95°C for 12 minutes followed by 35 cycles of 95°C for 20 seconds, 60°C for 30 seconds, 72°C for 30 seconds, and then a melting curve from 72°C to 90°C. The fluorescence was read after each cycle and every 0.2°C with a one second hold during the melt. Each genotype produced a product with a characteristic melting profile, as measured by T_m _of the negative first derivative of the disappearance of fluorescent signal. The Williams 82 homozygous genotype gave a peak at 83.6°C, mutant homozygous genotype gave a peak at 79.2°C, and a heterozygous genotype gave a peak at 83.6°C with a shoulder at 79.0°C. Templates for PCR were 1.2 mm washed FTA (Whatman, Clifton, NJ) card punches prepared from leaves according to the manufacturer's instructions

#### FAD2-1A allele-specific molecular marker assay

The TILLING screen originally identified line 18D as heterozygous for the S117N *FAD2-1A *mutation. Re-isolation of DNA from line 18D as well as line 16D and 17D M_2 _tissue and *FAD2-1A *sequence characterization revealed that line 18D (or 16D) did not contain the variant allele of *FAD2-1A*, but line 17D was homozygous for the S117N mutation. *FAD2-1A *specific gene amplification and sequencing was performed with the primers: 5'-accacctacttccacctccttcctcaa-3' and 5'-TATATGGGAGCATAAGGGTGGTAGTGGCTT-3'. The homozygous state of the S117N *FAD2-1A *allele in M_3 _seedlings from line 17D was also confirmed by sequencing.

An allele specific GC-tail molecular marker assay was developed for the S117N *FAD2-1A *allele identified in line 17D [34]. The primers used were: 5'-gcgggcagggcggcATCAACCCATTGGTACTTGC-3'; 5'-gcgccgATCAACCCATTGGTACTTGT-3'; and 5'-GTTGCCTTCTCACTGGTG-3'. Reactions were carried out in 15 μl; reaction conditions were the same as those in the *RS2 *allele-specific assay. PCR parameters on a DNA Engine Opticon 2 (MJ Research/Bio-Rad) for the *FAD2-1A *GC Tail assay were as follows: 95°C for 5 minutes followed by 35 cycles of 95°C for 20 seconds, 65°C for 20 seconds, 72°C for 20 seconds, and then a melting curve from 75°C to 85°C. The fluorescence was read after each cycle and every 0.2°C with a one second hold during the melt with excitation at 470–505 nm and detection at 523–543 nm.. Each genotype produced a product with a characteristic melting profile, as measured by T_m _of the negative first derivative of the disappearance of fluorescent signal. Homozygous wild-type *FAD2-1A *alleles produced a peak at 83°C, homozygous mutant alleles produced a peak at 81°C, and heterozygous samples produced both peaks. Templates for PCR were either genomic DNA samples isolated using the DNeasy Plant Mini Kit (Qiagen, Inc., Valencia, CA) or 1.2 mm washed FTA (Whatman) card punches prepared from leaves according to the manufacturer's instructions.

### Oligosaccharide Phenotype Determination

Oligosaccharides were determined by high performance ion chromatography with pulsed amperometric detection (PAD) employing an Agilent 1100 series HPLC and an ESA Coulochem III detector (Agilent Technologies, Chesterfield, MO, USA). A 12.5 mg ground seed sample from either a whole seed or a chipped seed (seed were cut with a razor blade so that approximately 1/2 could be used for oligosaccharide extract and the remaining half could be germinated) was extracted with 0.5 ml 50% ethanol at 70°C, 30 min. Samples were then centrifuged 15 min at 16,000 *g*. The supernatant was passed through a 0.2 μm filter. Sugars were separated on a Dionex Carbo Pac PA 10 analytical column (250 mm × 4 mm, 10 μm) connected to a Carbo Pac PA 10 guard column (50 mm × 4 mm). The mobile phase was 90 mM NaOH with flow rate of 1.5 ml min^-1^, maintained at 30°C. Detection settings were: time 0, 0.1 v, time 0.41, -2.0 v, time 0.42, 0.6 v, and time 0.44, -0.1 v.

### Fatty Acid Phenotype Determination

The fatty acid profiles of individual whole crushed seeds was determined by lipid gas chromatography of total fatty acid methyl esters of extracted oil, as previously described [33]. Five individual seeds from each of five plants were sampled from each line.

## Abbreviations

TILLING: Targeting induced local lesions in genomes; RS: raffinose synthase; FAD: fatty-acid desaturase; EMS: ethyl-methanesulfonate; NMU: N-nitroso-N-methylurea; SNP: single nucleotide polymorphism; PCR: polymerase chain reaction; NCBI: National Center for Biotechnology Information.

## Authors' contributions

ED designed and performed the raffinose synthase experiments and co-authored the manuscript. KB designed and performed the fatty acid desaturase experiments, guided the raffinose synthase experiments, and co-authored the manuscript. Both authors approved the final manuscript.
